# Free Radical Formation in the Reactions of Redox-Active Drugs and Xenobiotics with Mitochondrial Flavoenzymes

**DOI:** 10.3390/biom16060810

**Published:** 2026-05-29

**Authors:** Narimantas Čėnas

**Affiliations:** Institute of Biochemistry, Vilnius University, Saulėtekio 7, LT-10257 Vilnius, Lithuania; narimantas.cenas@bchi.vu.lt

**Keywords:** flavoenzymes, mitochondria, drugs, xenobiotics, redox cycling, oxidative stress

## Abstract

The single-electron reduction of redox-active drugs and xenobiotics (quinones, aromatic nitrocompounds, and *N*-oxides) by flavoenzymes, which initiates redox cycling and oxidative stress, is an important factor in their therapeutic/toxic effects. This review summarizes information on the action of mitochondrial flavoenzymes from various organisms in these processes, emphasizing the kinetic and mechanistic aspects. The flavoenzymes discussed also include those of which only a fraction is localized in mitochondria. According to kinetic data, the most effective generator of free radicals of xenobiotics is respiratory Complex I. However, it is unclear to what extent these reactions can compete with the rapid reduction of ubiquinone in normally functioning mitochondria. In specific cases, a very active free radical generator can be the NADPH:adrenodoxin reductase–adrenodoxin complex. The properties of other dehydrogenases–electrontransferases (succinate:ubiquinone reductase, fatty acid oxidation system) are less well characterized. Due to its high catalytic capacity, a potential but poorly studied source of free radicals of xenobiotics may be NADH:cytochrome *b*_5_ reductase and its complex with cytochrome *b*_5_. Flavoenzyme disulfide reductases, with the possible exception of *Plasmodium falciparum* thioredoxin reductase, are less active free radical generators. Importantly, in most cases, flavoenzymes perform the mixed single- and two-electron reduction of xenobiotics. According to the available data, the reactivity of redox cyclers depends mostly on their standard single-electron reduction potential and is little influenced by their structure. Therefore, in order to intensify these processes or achieve some structural specificity, it is necessary to focus on the selective accumulation of compounds in mitochondria.

## 1. Introduction

Flavoenzymes contain FMN (flavin mononucleotide) or FAD (flavin adenine dinucleotide) in their active sites and are responsible for many functions of the cell. From a mechanistic perspective, the uniqueness of this class of enzymes lies in the ability of many of them to switch from two-electron (hydride) transfer to the single-electron one, which is often reversible under physiological conditions, and frequently involves the flavin free radical (neutral or anionic semiquinone) intermediates [1]. In this context, when talking about single-electron transfer in flavoenzyme catalysis, one needs to consider the generation of reactive oxygen species (ROS, i.e., superoxide (O_2_^−•^), hydrogen peroxide (H_2_O_2_), and hydroxyl radicals (OH^•^), which is typically initiated by the single-electron reduction of oxygen by flavoenzymes [2]. In most cases, these reactions can be considered as secondary or side effects of these enzymes, which, however, often contribute to signal transduction in cells [3]. However, when ROS generation intensifies, i.e., goes beyond the regulatory limits of the cell antioxidant system, oxidative stress occurs, causing the modification of many essential biomolecules and subsequently cell death [4]. Most importantly, the antibacterial, antiparasitic, and sometimes antitumor effects of many redox-active compounds (quinones (Q), nitroaromatic compounds (ArNO_2_), and aromatic *N*-oxides (ArN → O)) are based on the mechanism of oxidative stress [5,6,7], which constitutes a decisive or significant part of their action. Alternatively, this may be the cause of the side effects of bactericidal or antiparasitic drugs, radiosensitizers, or environmental pollutants. In both cases, these compounds are reduced by flavoenzymes or by their physiological redox partners, metalloproteins, to their radicals, which are rapidly reoxidized by oxygen to form superoxide, which in turn induces oxidative stress (Equations (1) and (2)), where Q is quinone:

Q + e^−^ → Q^−•^, (1)

Q^−•^ + O_2_ ⇄ Q + O_2_^−•^ (2)

Historically, most oxidative stress-based cytotoxicity/therapeutic effects of the aforementioned compounds have been linked to their single-electron reduction by endoplasmic reticulum NADPH:cytochrome P450 reductase (P450R, EC 1.6.2.4) and its analogs, including NO synthase (NOS, EC 1.14.13.39) [8]. However, in this context, it has been noted that mitochondrial flavoenzymes have been somewhat overlooked, which may also contribute to oxidative stress or other types of toxicity of redox-active drugs or xenobiotics based on their bioreductive activation. In some sense, this is also linked to ROS-induced damage to mitochondrial DNA. Therefore, it should be noted that among the 77 flavoenzymes identified in human flavoproteome, 37 are localized in mitochondria, or in several locations within the cell, including mitochondria [9]. Among them, about 30 enzymes that mostly perform dehydrogenase and/or electron transferase functions could theoretically be responsible for the generation of ROS during their reactions with xenobiotics.

The aim of this review is to provide structured information on the catalysis mechanisms of mitochondrial flavoenzymes leading to the formation of free radicals of redox-active drugs or xenobiotics, and, where possible, the quantitative characterization of these reactions.

## 2. Single-Electron Transfer Properties of Prooxidant Drugs and Xenobiotics

**Figure 1:**
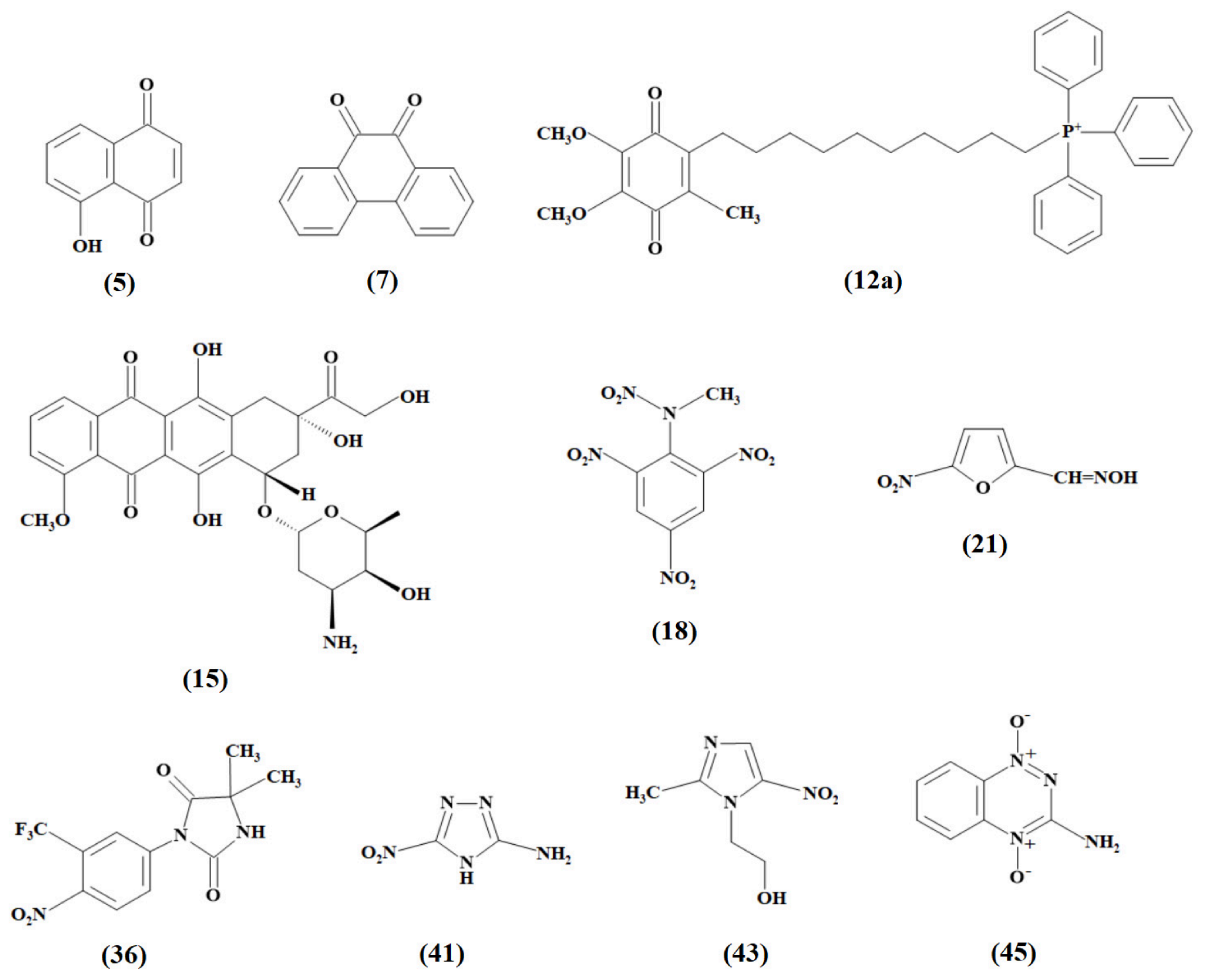
The formulas of the relevant representatives of prooxidant compounds: juglone (**5**), 9,10-phenanthrene quinone (**7**), mitoquinone (**12a**), adriamycin (**15**), tetryl (**18**), nifuroxime (**21**), nilutamide (**36**), ANTA (**41**), metronidazole (**43**), and tirapazamine (**45**). The number of compounds corresponds to those in Table 1. Mitoquinone is numbered as (**12a**) due to its similarity in redox properties to duroquinone (**12**).

In this section, the redox properties of prooxidant drugs and xenobiotics relevant to their possible redox cycling, and free radical and ROS formation in mitochondria are addressed. We will not analyze the problems associated with the enzymatic two-electron reduction of Q and ArNO_2_, and the reactions of ArN → O radicals under hypoxic conditions. In this context, it is necessary to separately discuss the factors determining the rates of enzymatic free radical formation of compounds and their oxidation by O_2_. The single-electron reduction of quinones, nitroaromatics, and aromatic *N*-oxides frequently follows an “outer-sphere” electron transfer model, which corresponds to an electron transfer with weak electronic coupling between the reactants ([10], and references therein). In the final expression, the rate constants of single-electron transfer between reagents (*k*_12_) will depend on the reactant electron self-exchange rate constants (*k*_11_ and *k*_22_) and the reaction equilibrium constant (*K*) (log *K* = Δ*E*^1^ (V)/0.059, where Δ*E*^1^ is the difference in the midpoint single-electron transfer potentials of reactants):

*k*_12_ = (*k*_11_ × *k*_22_ × *K* × *f*)^1/2^ (3)

and

log *f* = (log *K*)^2^/4 log (*k*_11_ × *k*_22_/*Z*^2^), (4)

where *Z* is a frequency factor (10^11^ M^−1^s^−1^). In the reaction of an electron donor with a series of homologous electron acceptors (*k*_22_ = constant), Equations (3) and (4) will give a parabolic (square) dependence of log *k*_12_ on Δ*E*^1^ with a slope Δlog *k*/ΔΔ*E*^1^ = 8.45 V^−1^ at Δ*E*^1^ = ±0.15 V. At Δ*E*^1^ = 0, *k*_12_ = (*k*_11_ × *k*_22_)^1/2^. For quinones and aromatic *N*-oxides, the self-exchange constants are around 10^8^ M^−1^s^−1^ [11,12]. For ArNO_2_, they are around 10^6^ M^−1^s^−1^ [13]. Since the midpoint potential of the single-electron reduction of oxidants is an important factor determining the rate of their reduction, Table 1 presents their values at pH 7.0 (*E*^1^_7_). Their values are determined mostly by pulse-radiolysis. The formulas of the relevant representatives of prooxidant compounds are presented in Figure 1.

Table 1 shows that most free radicals have a p*K*_a_ below 7.0, i.e., their formation from the oxidized compounds is likely to be a proton-unaccompanied electron transfer. A certain exception here would be nitroimidazoles and aromatic *N*-oxides, whose radical p*K*_a_ are between 4.7 and 6.2 ([16], Table 1). Further, the *E*^1^_7_ of newly synthesized or natural quinone compounds is reasonably predictable, based on the electron-accepting or donating properties of the substituents. The introduction of an alkyl, cycloalkyl, alkoxy, or aziridinyl group will decrease the *E*^1^_7_ of benzo- or naphthoquinone by 0.05–0.08 V almost additively. On the other hand, -OH groups forming intramolecular H-bonds with the carbonyl groups of 1,4-naphthoquinone or 9,10-anthraquinone will increase the *E*^1^_7_ by ~0.05 V (Table 1).

Regarding the reoxidation of the radicals of xenobiotics, the rate constants of these reactions may be described by Equations (3) and (4), taking −0.155 V for the *E*^1^_7_ of the O_2_/O_2_^−•^.couple, and its log *k*_22_ for 2.65 [20]. The reactivity of the free radicals of compounds decreases with increasing their *E*^1^_7_, so one can expect that their efficient free radical-initiated ROS generation will occur at their *E*^1^_7_ ≤ −0.1 V [5]. It follows from the data of Table 1 that the reoxidation of the radicals of nitroaromatics and aromatic *N*-oxides is practically irreversible and leads to their prooxidant action. On the other hand, the prooxidant action of quinones with higher *E*^1^_7_ or unstable semiquinone may be complicated by the rapid dismutation of their radicals, leading to the formation of antioxidant hydroquinones Equation (5):

2 Q^−•^ + 2 H^+^ ⇄ Q + QH_2_ (5)

This phenomenon is very important in the development of new quinones with both anti- and prooxidant properties, such as mitoquinone (**12a**) or its 10-(6’-plastoquinonyl)-analogs, with the antioxidant effect of the latter in rat heart mitochondria being manifested in a wide concentration range of 10^−8^–10^−6^ M [21]. However, antioxidant properties are most characteristic of a limited group of benzo- or naphthoquinones, which are fully or highly substituted with electron-donating groups [22]. In general, the antioxidant effects of quinones, or rather their reduced forms, associated with ROS scavenging, occur at lower concentrations than their prooxidant effects, which strongly depend on the system under study and the incubation time [23]. However, a more detailed analysis of this problem is beyond the scope of this review.

## 3. Flavoenzymes Participating in the Formation of Free Radicals of Prooxidant Drugs and Xenobiotics in Mitochondria

### 3.1. Mitochondrial Concentrations of Flavoenzymes and Their Physiological Redox Partners Capable of Generating Free Radicals from Drugs and Xenobiotics

In order to quantitatively understand the role of mitochondrial flavoenzymes as sources of xenobiotic free radicals, in addition to their kinetic characteristics, it is necessary to know their amount in mitochondria. However, this information, both concerning relevant flavoproteins (their properties are discussed in the following sections) and their physiological redox partners, metalloproteins, is scarce (Table 2).

It should also be noted that the concentrations of flavoenzymes vary significantly between species and organs (Table 2). Also, to the best of our knowledge, the amount of cytochrome *b*_5_ reductase in mitochondria has not been determined, but based on the data of microsomes, it can be expected to be an order of magnitude lower than cytochrome *b*_5_ (Table 2).

In the following sections, we will review the catalytic properties of a number of mitochondrial flavoenzymes and their reactions with prooxidant drugs and xenobiotics that can generate free radicals in mitochondria. The redox potentials of the flavoenzymes, reflecting their reducing potency in both single- and two-electron transfer reactions, are presented in the text and summarized in Appendix A.

### 3.2. NAD(P)H-Oxidizing Flavoenzymes Dehydrogenases–Electrontransferases

Flavoenzymes dehydrogenases–electrontransferases switch from two-electron (hydride) transfer to a single-electron one, and typically have a single-electron transferring redox partner, heme- or FeS-containing protein. Their action frequently involves the formation of neutral (blue) flavin semiquinone, FMNH^•^ or FADH^•^, as an intermediate reaction product.

Mammalian NADH:ubiquinone oxidoreductase (Complex I, EC 1.6.5.3), consisting of 45 subunits, is localized in the inner mitochondrial membrane. It catalyzes NADH oxidation by ubiquinone and performs transmembrane two-proton translocation for each electron transferred. In bovine L-shaped Complex I, FMN and 8 FeS clusters N1-6 are localized in the hydrophilic arm directed to the matrix ([31], and references cited therein). FMN is located in the 51 kD subunit. The redox equivalents are transferred in a sequence: NADH → FMN (*E*_7.5_ (FMN/FMNH^•^) = −0.415 V, *E*_7.5_(FMNH^•^/FMNH^−^) = −0.336 V [32]) → N3 (N1a) →N1b → N4 → N5 → N6a → N6b → N2 → bound ubiquinone. The *E*^1^_7_ of N1a is below −0.380 V, the *E*^1^_7_ values of N1b, N3, N4, and N5 are in the range of −0.240–−0.270 V, and the *E*^1^_7_ of N2 is in the range of −0.050–−0.120 V [33]. In proteoliposomes, the maximal rate of ubiquinone reduction determined in steady-state reactions is 150–380 s^−1^ [34]. The reduction of ubiquinone is inhibited by rotenone and piericidin with nanomolar inhibition constants.

The Complex I-mediated redox cycling of adriamycin (**15**) was reported in the 1980s [35]. Adriamycin restored rotenone-inhibited NADH oxidation in bovine heart submitochondrial particles and induced ROS generation [35]. It was suggested that adriamycin binds to FMN at the NAD^+^ binding site because it inhibited NAD^+^ reduction by succinate during reverse electron transfer. On the other hand, this raises doubts as to whether these processes can occur during natural mitochondrial function, i.e., whether part of the electron flux can be directed to external oxidants, thereby competing with the rapid reduction of ubiquinone. However, this possibility was later confirmed because other quinones stimulated ROS formation during the oxidation of the NAD-dependent substrates glutamate and malate in rat brain, liver, and heart, and in bovine aortic endothelial mitochondria, even in the absence of rotenone [36,37,38,39].

Typically, the reduction of hydrophilic quinones, ArNO_2_, and inorganic complexes by isolated Complex I is partly inhibited or not inhibited by rotenone. In the case of quinones, sensitivity increases in the presence of phospholipids in the medium, thus pointing to a partial involvement of FeS cluster N2 [40]. One may note that the mechanism(s) of rotenone-insensitive reduction of electron acceptors are still a matter of debate, partly due to different experimental conditions. In this case, the most thoroughly studied reaction is the reduction of ferricyanide, where ferricyanide presumably directly oxidizes reduced FMN [41,42]. This reaction is characterized by a “ping-pong” mechanism with *k*_cat_ ≥ 5000 e^−^/s, and also demonstrates double competitive substrate inhibition, i.e., the competition of both substrates for the same binding site in reduced and oxidized enzyme form. Since inhibition by both substrates decreases with increasing ionic strength, this indicates that they interact with a positively charged group [42]. The use of 4-*S*-NADD decreases the *k*_cat_ of the reaction and the *k*_cat_/*K*_m_ of NADH two times, thus showing that the rate-limiting step of the process is the reduction of FMN by NADH. On the other hand, the positively charged hexaammine ruthenium (III) (Ru(NH_3_)_6_^3+^) and possibly dimethyl viologen are reduced by a “ternary complex” mechanism, i.e., they can only oxidize complexes of reduced FMN with NAD(H) [43,44]. These reactions are not inhibited by NADH [44]. However, a strong (*K*_i_ ≈ 10^−8^ M) competitive to the NADH inhibitor, NADH-OH (a 2,6-dihydroxynicotinamide derivative), does not inhibit the reduction of Ru(NH_3_)_6_^3+^ by succinate during reverse electron transfer in submitochondrial particles, suggesting that FMN is not involved in its reduction [45].

In structure–activity relationship studies, the reactivity of soluble model quinones, including ubiquinone-1 and adriamycin (**15**) and nitroaromatics towards bovine heart Complex I, follows a common parabolic dependence of log *k*_cat_/K_m_ on the *E*^1^_7_ of oxidants [46]. The *k*_cat_ of the reaction reaches 100 s^−1^, and *k*_cat_/*K*_m_ reaches 8.0 × 10^5^ M^−1^s^−1^ for the most active oxidants. Using 2-(3-hydroxy-3-methyl-butyl)-3,5,6-trimethyl-1,4-benzoquinone as an oxidant, it was shown that the *k*_cat_/*K*_m_ of its reduction by Complex I inside bovine mitochondria is about 20 times lower than that of the cytochrome P450 reductase inside rat liver microsomes [47]. The oxidants are reduced in a mixed single- and two-electron way, with a single-electron flux of 25% (1,4-benzoquinone (**1**)), ~50% (hydroxynaphthoquinone pigments [48]), and 45–70% (ArNO_2_) [46]. Another study also showed that Complex I catalyzes the oxidation of excess NADH by hydrophilic ubiquinone derivatives, indicating that their redox cycling occurs [40]. The studies of reaction inhibition by NADH, NAD^+^, redox inactive ADP-ribose, and slowly reacting quinones led to the conclusion that quinones and ArNO_2_ bind close to the ferricyanide and NAD(H) binding site [46,48,49]. In contrast to ferricyanide, they may oxidize both the free enzyme form and its complexes with NADH (*K*_d_ = 3.0 µM) and NAD^+^ (*K*_d_ = 30–60 µM), although with 2.4 and 7.2 times lower *k*_cat_/*K*_m_, respectively. Importantly, these *K*_d_ are about ten times lower than the corresponding inhibition constants in ferricyanide reduction determined under identical conditions [42,49]. Therefore it is possible that in the rate-limiting step of oxidative half-reaction, ferricyanide and quinones or ArNO_2_ react with different redox states of FMN with different affinities for NAD(H).

Of particular interest are studies of the redox cycling of the mitochondria-targeted compounds mitoquinone (10-(6’-ubiquinonyl)decyltriphenylphosphonium (**12a**)) [38] and alkyltriphenylphosphonium-substituted 4,4’-bipyridinium derivatives, e.g., mitoparaquat [39], which are attributed to Complex I action. The presence of alkyltriphenyl–phosphonium groups enhances the accumulation of these compounds in the matrix and intensifies ROS generation.

*Trypanosoma brucei* NADH:ubiquinone oxidoreductase (m.w. ~600 kD, 11 subunits) contains a 33 kD subunit with bound FMN, which is analogous to the 51 kD FMN-binding subunit of bovine Complex I [50]. The dimerized form of a 33 kD subunit was purified, and it was shown that it reduces 2,3-dimethoxy-5-methyl-1,4-benzoquinone and low-m.w. ubiquinones in a single-electron way; however, the kinetics of these reactions have not been detailed [51].

NADPH:adrenodoxin reductase (ADR, EC 1.18.1.6) is localized in the inner mitochondrial membrane and the matrix. It is a monomeric 51 kD FAD-containing enzyme, which was first isolated from bovine adrenal cortex mitochondria. The enzyme is also expressed in the liver, the kidney, and the placenta. The thermodynamic and kinetic properties of bovine adrenal cortex enzyme are summarized below [52,53,54,55,56,57]: (i) ADR (*E*^0^_7_ = −0.250–−0.274 V, *E*_7_ (FAD/FADH^•^) = −0.320 V) reduces adrenodoxin (ADX), a 13.3 kD Fe_2_S_2_ protein (*E*^1^_7_ = −0.260 V in a free form, and *E*^1^_7_ = −0.360 V in ADR-bound form), which in turn reduces mitochondrial cytochromes P-450_scc_ and P450_11β_ that participate in the biosynthesis of steroid hormones; (ii) in a reductive half-reaction, NADPH reduces ADR with *k* = 28 s^−1^ with the formation of the FADH_2_-NADP^+^ complex (*K*_d_ = 10^−8^ M). The binding of NADP^+^ to the oxidized and semiquinone form of ADR is characterized by a lower affinity. NADPH binds to the FAD semiquinone more efficiently than NADP^+^, and (iii) the ADR-ADX complex formation is governed by the electrostatic interaction between the positively charged amino acid residues of ADR and the negatively charged residues of ADX. At ionic strengths of 0.1–0.2 M, the *K*_d_ of this complex is in the submicromolar range, and ADR reduces ADX with *k*_cat_ = 10 e^−^/s.

**Scheme 1:**
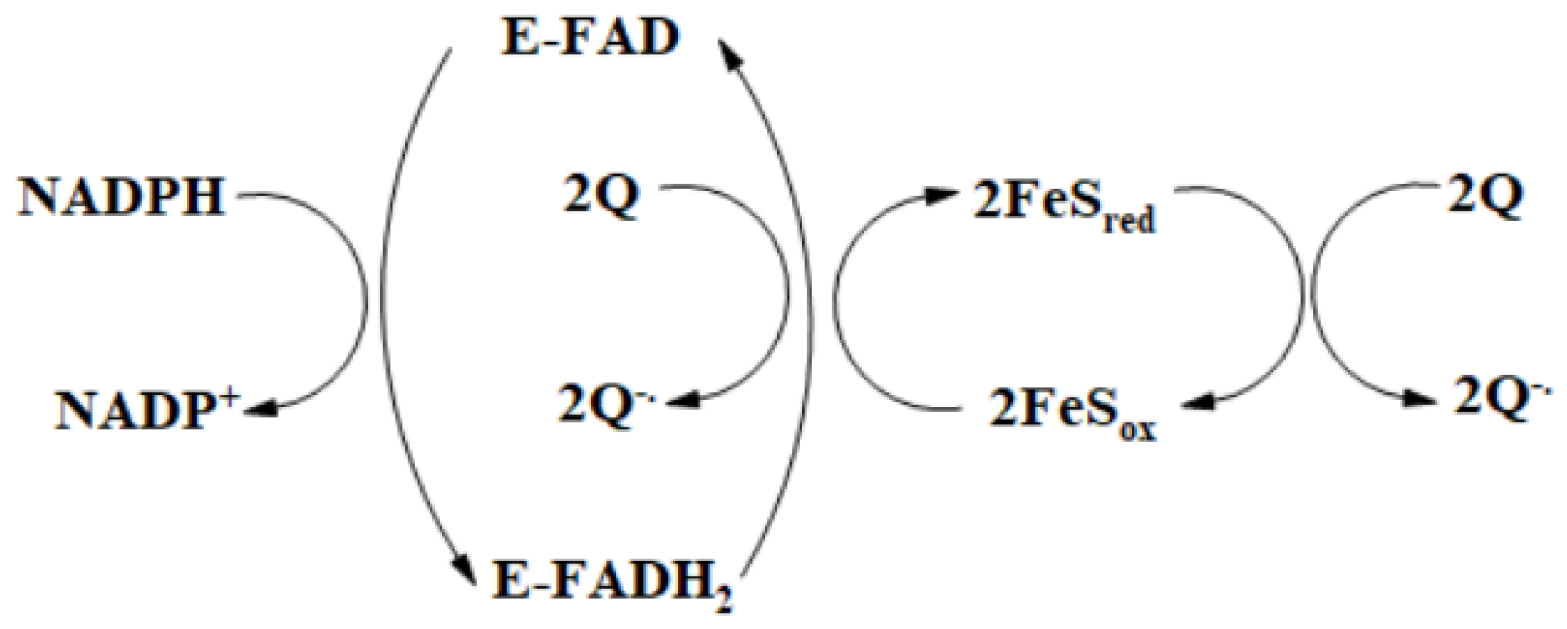
A scheme of the reduction of quinones (Q) by ADR or other FeS reductases and their FeS redox partners.

**Figure 2:**
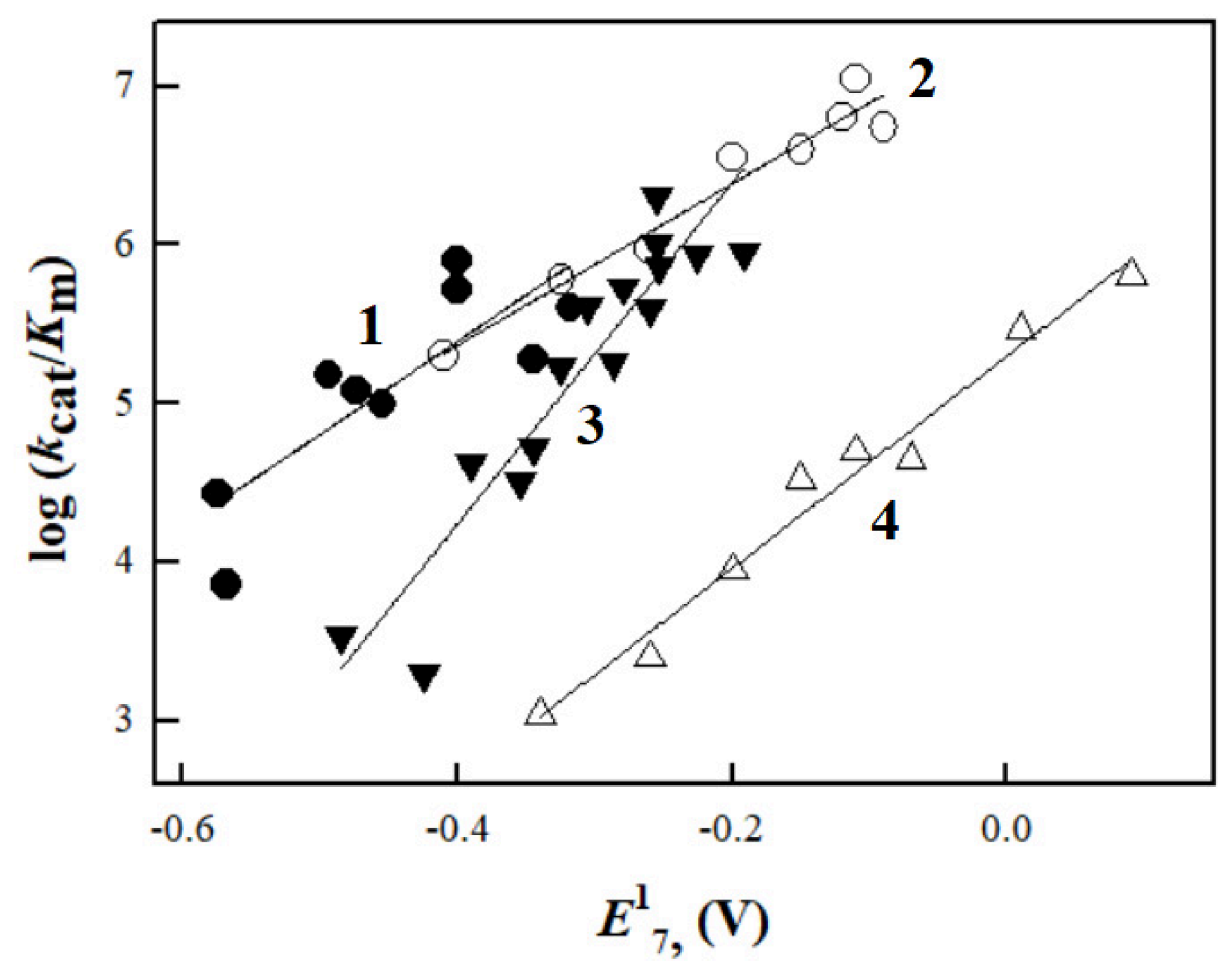
The dependence of the reactivity (log *k*_cat_/*K*_m_) of ArN → O (1), Q (2,4), and ArNO_2_ (3) in adrenodoxin reductase-catalyzed reduction in the presence of adrenodoxin (1,2,3) and its absence (4). Data taken from [58,59,60,61].

In steady-state reactions, ADR reduces quinones with *k_cat_* = 20–25 s^−1^, which is close to 28 s^−1^, the maximal rate of enzyme reduction by NADPH. However, NADPH is a strong (*K*_i_ = 5.0–6.0 µM) but incomplete inhibitor with respect to oxidants. Obtained by extrapolation to [NADPH] = 0, *k*_cat_/K_m_ values for the reduction of quinones vary from 1.0 × 10^3^ M^−1^s^−1^ to 5.0 × 10^5^ M^−1^s^−1^, increasing with an increase in their *E*^1^_7_ (Figure 2) [58,59]. 1,4-Benzoquinone is reduced with 50% single-electron flux. ADR possesses low nitroreductase activity; the *k*_cat_/K_m_ of nitrofurans at [NADPH] = 0 are in the range 1.0–4.0 × 10^3^ M^−1^s^−1^ [60]. It can be assumed that Q and ArNO_2_ oxidize FADH^•^ and its complexes with NADPH and NADP^+^ at the rate-limiting step, while free ADR is oxidized more rapidly than its complexes. Importantly, ADX stimulates the reduction of Q, ArNO_2_, and ArN → O by ADR, eliminating the inhibition by NADPH and providing a more efficient ADX-mediated reduction pathway [12,61] (Scheme 1).

The reaction proceeds with *k*_cat_ = 8–9 e^−^/s, i.e., close to the maximal rate of ADX reduction by ADR. The single-electron character oxidant reduction is confirmed by the stoichiometric oxidation of NADPH and O_2_ consumption, as well as the oxidation of a significant excess of NADPH with respect to the oxidant. ADR reactivity data in the absence and presence of ADX are summarized in Figure 2. It shows that the reactivity of quinones in the ADX-mediated reduction is two orders of magnitude higher than in the case of ADR alone, and that in the ADX-mediated reduction, ArNO_2_ are less reactive than Q and ArN → O, which, according to Equation (1), is determined by the much lower *k_22_* of nitroaromatics.

NADH:cytochrome *b*_5_ reductase (b5R, EC 1.6.2.2) and its physiological oxidant, cytochrome *b*_5_, are located both in the endoplasmic reticulum and the inner side of the outer mitochondrial membrane. Both forms of b5R are identical and encoded by the same gene [62]. B5R is a monomeric 33 kD FAD-containing protein. The best characterized b5R from rat liver at pH 7.0 is reduced by NADH during the dead-time of the stopped-flow experiments with the formation of the FADH^−^–NAD^+^ charge-transfer complex [63]. In steady-state reactions at pH 7.0, rat b5R reduces cytochrome *b*_5_ with *k*_cat_ = 600 s^−1^, and ferricyanide with *k*_cat_ = 800 s^−1^ [64]. FADH^•^ is formed as an unstable intermediate in the oxidation of the reduced enzyme by ferricyanide [65]; however, no semiquinone is formed when titrating b5R with dithionite, which gives the FAD an *E*^0^_7_ of −0.258 V [66]. On the other hand, the presence of NAD^+^ during the titration shifts the *E*^0^_7_ of the enzyme in a positive direction, and stabilizes the anionic FAD semiquinone. In this case, *E*_7_(FAD/FAD^−•^) is equal to −0.088 V, and *E*_7_(FAD^−•^/FADH^−^) is equal to −0.147 V [63]. This is caused by a preferential binding of NAD^+^ to the reduced and semiquinone forms of enzymes. The discrepancy between the FADH^•^ and FAD^−•^ formation was explained by the fact that FADH^•^ possesses a p*K*_a_ = 6.3, and is rapidly deprotonated at neutral pH [67].

After some debate, mitochondrial b5R was identified with "external" NADH dehydrogenase of the outer mitochondrial membrane [68]. Although the single-electron reduction of quinones by b5R was documented around the 1970s [69], these reactions, together with the reduction of methyl viologen (paraquat) and nitrofurantoin [70,71,72], were not clearly characterized by kinetic parameters. At a fixed quinone concentration, the rate of b5R-catalyzed NADH oxidation increased with an increase in the *E*^1^_7_ of quinones [70]. A later more detailed study estimated that in rat microsomes, b5R reduces 2-tocopherylquinone and ubiquinone-3 (expected *E*^1^_7_ = −0.24 V) with *k*_cat_ = 5.1 s^−1^ and *k*_cat_/*K*_m_ = 1.1 × 10^5^ M^−1^s^−1^, and with *k*_cat_ = 53 s^−1^ and *k*_cat_/*K*_m_ = 5.0 × 10^5^ M^−1^s^−1^ [47], respectively. These values are slightly higher than those obtained for mitochondrial Complex I in bovine submitochondrial preparations [47].

The microsomal and mitochondrial forms of the physiological oxidant of b5R, cytochrome *b*_5_, are encoded by different genes. Therefore, the protoheme-IX surroundings of these proteins are different [73]. This results in the changes in their *E*^1^_7_ from 0.01 V (rat microsomes) to −0.107 V (rat mitochondria) and −0.04 V (human mitochondria) [74]. In rat liver mitochondria, cytochrome *b*_5_ efficiently reduces cytochrome *c*, which further passes electrons to cytochrome *c* oxidase, thus performing the rotenone-insensitive oxidation of exogenous NADH [75]. However, it is not definitively understood whether and how cytochrome *b*_5_ is involved in the reduction of quinones and other drugs or xenobiotics, as previous studies have been performed in the presence of b5R. It can be calculated that isolated microsomal cytochrome *b*_5_ reduces menadione (**10**) (*E*^1^_7_ = −0.20 V) with *k* = 10^4^ M^−1^s^−1^ [76], i.e., it is slower than the above-mentioned rate constants of b5R [47]. However, one might expect these reactions to be faster due to the lower redox potential of mitochondrial cytochrome *b*_5_. On the other hand, cytochrome *b*_5_ stimulates quinone reduction by b5R [76]. In addition to its possible direct involvement, as in the case of ADR/ADX [12,61], it has been shown that the formation of a b5R complex with cytochrome *b*_5_ decreases the *E*^0^_7_ of FAD by 0.04 V [77]. This may increase the reactivity of b5R towards quinones and other redox-active compounds.

### 3.3. Flavoenzyme Disulfide Reductases

Flavoenzyme disulfide reductases, in most cases, perform antioxidant functions that participate in the common thiol antioxidant system of the cell. They contain FAD and redox-active disulfide groups per subunit, which transfer two redox equivalents in a sequence, NAD(P)H → FAD → catalytic disulfide → low-Mr, or protein disulfide substrate. These reactions do not involve the formation of free radical intermediates ([78], and references therein). On the other hand, their distinguishing feature is the formation of an oxidized FAD-reduced disulfide charge-transfer complex absorbing at 530 nm in the 2e^−^ -reduced form (EH_2_). Although these enzymes typically possess low to moderate quinone or nitroreductase activity (see below), they are often considered important targets for redox cycling of antiparasitic or antitumor agents [79]. Therefore, their above-mentioned reactions deserve detailed analysis.

Glutathione reductase (GR, EC 1.8.1.7), the 2 × 55 kD homodimer, is the most well-described representative of this group. In humans, the mitochondrial and cytosolic (erythrocyte) GR isoenzymes are encoded by the same gene, and, with the exception of an arginine-rich 44-amino acid extension at the *N*-terminal, its amino acid sequence is identical to erythrocyte GR [80]. Mitochondrial GR is located in the matrix. The ratio of mitochondrial to cytosolic GR content in mammalian cells is 8–24% [81,82]. Therefore, in the absence of sufficient data on the properties of the mitochondrial enzyme, it can be expected that they will be the same as those of erythrocyte GR. This enzyme is reduced by NADPH to EH_2_, and EH_2_ is reoxidized by glutathione (GSSG), with both substrates binding at different sites. In a “hybrid ping-pong” mechanism (GSSG reoxidizes not free EH_2_ but its complex with repeatedly tightly bound NADPH), the oxidative half-reaction is a rate-limiting step [83]. The enzyme *k*_cat_ value is equal to 120 s^−1^; its two-electron reduction potential is equal to −0.227 V at pH 7.0 [84]. The four-electron reduced state (EH_4_) of GR is not formed in the catalytic cycle of the enzyme. Erythrocyte GR relatively slowly reduces quinones (*E*^1^_7_ = −0.10–−0.24 V) in a mixed single- and two-electron way with *k*_cat_ = 0.11–4.0 s^−1^ and *k*_cat_/*K*_m_ = 2.7 × 10^2^–4.0 × 10^4^ M^−1^s^−1^ [85,86]. Among the nitroaromatic compounds that are reduced in a single-electron way, the most efficient oxidant of GR is tetryl (**18**) (*k*_cat_ ≥ 5 s^−1^, *k*_cat_/*K*_m_ = 2.0 × 10^3^ M^−1^s^−1^ [87]); however, most nitrobenzenes and nitrofurans were almost inactive as GR oxidants, with the *k*_cat_ not exceeding 0.1 s^−1^ [88]. Importantly, quinone reductase reactions of GR are activated by the reaction product NADP^+^ [89]. Although the main electron density in the FAD–thiolate charge-transfer complex is localized on thiolate, its minor part remains on FAD. NADP^+^, when bound to EH_2_, replaces NADPH and increases the electron density of FAD, which may accelerate the reduction in xenobiotics. This demonstrates the participation of reduced FAD in the reduction of quinones and other redox cyclers.

Lipoamide dehydrogenase (LipDH, NADH:lipoamide oxidoreductase, EC 1.8.1.4) is structurally similar to GR [79]. Mammalian LipDH is a component of the pyruvate dehydrogenase or α-ketoglutarate dehydrogenase complexes in mitochondria, which are localized in the matrix and specifically associated with the inner mitochondrial membrane. The LipDH catalyzes the NAD^+^-dependent oxidation of covalently bound dihydrolipoate. The most studied pig heart enzyme catalyzes the rapid and reversible transfer of two redox equivalents from dihydrolipoamide to NAD^+^ and from NADH to lipoamide (LipS_2_NH_2_). In the latter case, the rate-limiting step of the process is the oxidative half-reaction [90]. In catalysis, LipDH shuttles between E_ox_ and EH_2_ states; the *E*^0^_7_ values for E_ox_/EH_2_ and EH_2_/EH_4_ redox couples are −0.280 V and −0.345 V, respectively [91]. When studying model quinones, it was found that pig heart LipDH reduces them with *k*_cat_ = 70 s^−1^, i.e., 17% of the *k*_cat_ of LIpS_2_NH_2_ reduction. In this case, the rate-limiting step is the enzyme reduction by NADH. The *k*c_at_/*K*_m_ for quinone reduction varies from 1.3 × 10^6^ M^−1^s^−1^ to 2 × 10^2^ M^−1^s^−1^, and increases with increasing quinone *E*^1^*_7_*, i.e., is not structure-specific [92]. LipDH reduces quinones with a 15–20% single-electron flux. It was concluded that quinones are mostly reduced via reduced FAD by the fraction of EH_4_, which is formed during the reduction of LipDH by NADH in vitro in the absence of NAD^+^, due to the favorable redox potential of the EH_2_/EH_4_ couple. However, when the redox potential of the medium corresponded to the functioning of EH_2_ only ([NAD^+^]/[NADH] = 4.7), the *k*_cat_/*K*_m_ of quinone reduction decreased by 5–10 times and became similar to these in GR-catalyzed reactions [93]. Under these conditions, the single-electron flux increases up to 30%. Pig heart LipDH reduces nitroaromatic compounds slowly, with *k*_cat_ = 0.1 ÷ 2.0 s^−1^ in a single-electron way [94].

To the best of our knowledge, the reactions of human LipDH with redox-active drugs and xenobiotics have not been examined so far. Another relatively well-studied representative of this enzyme group is LipDH from *Trypanosoma cruzi*; the latter possesses a single, highly specialized mitochondrion that undergoes extensive remodeling during their life cycle. For this reason, the localization of LipDH in the mitochondrion was confirmed only after a long debate [95]. LipDH is essential for both bloodstream and procyclic forms of *T. brucei* [95]. *T. cruzi* LipDH is a 2 × 55 kD homodimer [96]; its amino acid sequence is 81% identical to that of *T. brucei*, but only 50% identical to the human LipDH [97]. Its *k*_cat_ using LipS_2_NH_2_ as the oxidant is 107 s^−1^ [98]. *T. cruzi* LipDH reduces menadione (**10**) (*E*^1^_7_ = −0.20 V) and plumbagin (**9**) (*E*^1^_7_ = −0.16 V), with *k*_cat_ = 17 s^−1^ and *k*_cat_/*K*_m_ = 10^5^ M^−1^s^−1^, and *k*_cat_= 19 s^−1^ and *k*_cat_/*K*_m_ = 4.8 × 10^5^ M^−1^s^−1^, respectively [99]. Their 3-alkyl derivatives, presumably possessing lower *E*^1^_7_ values, are reduced several times slower. These reactions are characterized by 47–80% single-electron flux. In parallel experiments, pig heart LipDH reduces these compounds at similar rates. In another study, *T. cruzi* LipDH reduced methylene blue (*E*^1^_7_ = −0.10 V) at higher rates, *k*_cat_ = 45 s^−1^ and *k*_cat_/*K*_m_ = 8.5 × 10^5^ M^−1^s^−1^ [98], but reduced nitrofurans (*E*^1^_7_ ~ −0.25 V) very slowly, with *k*_cat_ = 0.1 ÷ 2.0 s^−1^ [88]. Taken together, these data suggest that, as in the case of the pig heart enzyme, the quinone reductase activity of *T. cruzi* LipDH is determined more by the quinone reduction potential than by its structural features.

Mammalian NADPH:thioredoxin reductases (TrxRs, EC 1.8.1.9) are 2 × 58 kD homodimers that contain FAD, catalytic disulfide, and a third cofactor, selenylsulfide, in their active center. The latter is located at the C-end of the protein in the conserved sequence Gly–Cys–SeCys–Gly. Mammalian cells contain three isoforms of TrxR in the cytosol (TrxR1), mitochondria (TrxR2), and testes (TrxR3), which are encoded by three different genes [100,101]. The physiological substrates of TrxRs are 10–12 kD disulfide proteins, thioredoxins (Trxs), which perform antioxidant and other numerous physiological functions. Among the three enzyme isoforms, the mechanism of TrxR1 catalysis is the best studied so far. During catalysis, TrxR1 cycles between EH_2_ and EH_4_ redox states, where the main electron density is located on dithiolate and reduced selenylsulfide. The reduced TrxR form is the typical FAD–thiolate charge-transfer complex with λ_max_ = 530 nm. The rate-limiting catalysis step of TrxR1 is the intramolecular two-electron transfer between dithiolate and selenylsulfide [102], which is characterized by *k*_cat_ = 30–40 s^−1^. It can be assumed that this rate-limiting step is also characteristic of TrxR2, but it is 1.5–3.0 times slower [103]. The *E*^0^_7_ for the EH_2_/EH_4_ redox couple of human TrxR2 is −0.270 V, which is more positive than that of human and rat TrxR1, −0.295 V [104,105]. Because TrxRs reversibly react with the NADP^+^/NADPH couple, the higher *E*^0^_7_ may enable TrxR2 to exert its antioxidant functions at higher [NADP^+^]/[NADPH] ratios in mitochondria than in cytosol [106]. The higher redox potential of TrxR2 can be determined by the presence of basic His125 and His-488 residues, instead of Tyr116 and His472 in rat and human TrxR1 in the vicinity of reduced disulfide–selenylsulfide [101]. The presence of additional histidine may stabilize the EH_4_ state of TrxR2 and increase its oxidation potential.

Like rat and human TrxR1, human TrxR2 reduces quinones and nitroaromatic compounds in a predominantly mixed single- and two-electron way with the *k*_cat_ values of 11.0–0.4 s^−1^ and the *k*_cat_/*K*_m_ values of 8.8 × 10^5^–3.0 × 10^2^ M^−1^s^−1^ [104]. There is a certain trend for reactions to accelerate with increasing *E*^1^_7_ of compounds. As a rule, these reactions are 2–3 times slower than in the case of human or rat TrxR1 [104,105,107]. By analogy with TrxR1, it can be assumed that, in parallel to reduced FAD, reduced selenylsulfide is also involved in these reactions, especially in the reduction of fully substituted quinones [105,107]. The participation of selenocysteine but not cysteine in these reactions may be explained by its lower single-electron oxidation potential since *E*_7_(Sec^•^/Sec^−^) is equal to 0.43 V, whereas *E*_7_(Cys^•^/CysH) is equal to 0.92 V [108]. A separate problem in this context is the NADPH oxidase reaction of TrxRs with the formation of superoxide, which occurs when the enzyme reacts with alkylating agents that attack reduced catalytic selenylsulfide and/or disulfide, including 1-chloro-2,4-dinitrobenzene, tetryl, Tri-1, and partly substituted quinones [107,109,110]. This covalent modification is accompanied by a loss of activity against physiological oxidants, with the maximum rate of the NADPH:oxidase reaction of TrxR1 being 0.10–1.5 s^−1^ [110]. It can be expected that in the case of TrxR2, these activities will be 2–3 times lower.

Among other mitochondrial TrxRs, *P. falciparum* TrxR (*Pf*TrxR) is the most studied for its reactions with redox cycling agents. This enzyme shares only 40% sequence analogy with human TrxR2 [111]. However, the most important difference is that the C-terminal catalytic groups Cys522-Sec523 in human TrxR2 are replaced by Cys535-Cys540 in *Pf*TrxR [112]. This catalytic disulfide receives electrons from the redox-active Cys88-Cys93 at the N-terminal, which in turn receives electrons from the reduced FAD. The reduction of FAD by NADPH occurs very rapidly, with *k* = 720–480 s^−1^, whereas the reduction of *P. falciparum* thioredoxin, presumably via the *C*-terminal dicysteine, is much slower, 20–29 s^−1^ [112]. The redox potential of *Pf*TrxR has not been determined, but it may be close to the *E*^0^_7_ of the *Drosophila melanogaster* TrxR EH_2_/EH_4_ couple, −0.298 V, as the *E*^0^_7_ of the physiological oxidants are very close, −0.275 V (*D. melanogaster* Trx) and −0.272 V (*P. falciparum* Trx) [113]. *Pf*TrxR reduces 1,4-naphthoquinone (*E*^1^_7_ = −0.15 V) with *k_cat_* = 12.5 s^−1^ and *k*_cat_/*K_m_* = 1.8 × 10^5^ M^−1^s^−1^, and menadione (*E*^1^_7_ = −0.20 V) with *k*_cat_ = 30.7 s^−1^ and *k*_cat_/*K*_m_ = 1.6 × 10^5^ M^−1^s^−1^ [86]. Aza-naphthoquinones, most likely having larger or similar *E*^1^_7_ as 1,4-naphthoquinones, are reduced at similar or higher rates, with *k*_cat_ varying from 67 s^−1^ to 6.2 s^−1^, and *k*_cat_/*K*_m_ varying in the range of 3.9 ÷ 1.1 × 10^5^ M^−1^s^−1^ [86]. Since the *k*_cat_ of these reactions often exceeds the maximal rate of thioredoxin reduction, it can be argued that, as in similar cases, quinones are reduced by reduced FAD. On the other hand, eosin B, which has a much lower reduction potential (*E*^1^_7_ ≤ −0.60 V [114]) is reduced much more slowly, with *k*_cat_ = 0.11 s^−1^ and *k*_cat_/*K*_m_ = 10^4^ M^−1^s^−1^ [115]. As in other cases, these data also demonstrate the positive influence of the reduction potential on the reactivity of compounds, although the influence of their structure remains insufficiently revealed.

Summing up, the single- or mixed single- and two-electron reduction of quinones or other redox agents by flavin disulfide reductases is somewhat unexpected, as these enzymes perform the obligatory two-electron (hydride) reduction of their physiological oxidants, disulfides. However, the former reactions involve not catalytic disulfides but FAD, whose single-electron reduction energetics are not characterized so far. On the other hand, the formation of anionic FAD semiquinone upon reduction of GR by deazaflavin radicals [116], and the paramagnetic character of the FADH_2_-NADP+ charge-transfer complexes of GR and LipDH [79], support the possibility of single-electron transfer. Another important point is the inhibition of disulfide reductases by electron-deficient aromatic compounds, including quinones and nitroaromatics [79]. The best studied is the binding of these compounds to the cavity in the intersubunit domain of GR, with the participation of His75,82, Phe78, and Tyr407 [117]. This is also possible in the case of mammalian and *P. falciparum* TrxR, which also have such cavities [118,119]. This is not the site of reduction for these redox-active compounds because it is too distant from the isoalloxazine ring. However, inhibition of these antioxidant enzymes may increase the effects of oxidative stress on the cell.

### 3.4. Other NAD(P)H-Oxidizing Flavoenzymes

The alternative type-2 FAD-containing NADH:ubiquinone oxidoreductases (NDH-2s, EC 1.6.5.9) are found in the mitochondria of various eukaryotic organisms, e.g., plants, yeast, and protists, but not in mammalian mitochondria [120]. These enzymes lack a transmembrane domain and are not proton-pumping; moreover, they are composed of a single polypeptide chain. Another important feature is that, despite the "ping-pong" reaction mechanism, the NADH nicotinamide ring and ubiquinone bind in separate sites at the isoalloxazine ring of FAD [120,121]. For NDH-2s, the *E*^0^_7_ values, being equal to −0.220 V, are determined only for *Staphylococcus aureus* and *Escherichia coli* enzymes [122,123]. Among these enzymes, the reactions of *P. falciparum* NDH-2 with various groups of redox agents have been studied in the most detail [124]. This enzyme reduces quinones and nitroaromatic compounds with *k*_cat_ ranging from 41 to 0.4 s^−1^ and *k*_cat_/*K*_m_ ranging from 2.8 × 10^6^ to 250 M^−1^s^−1^. These compounds oxidize complexes of the reduced enzyme with NADH and NAD^+^, both of which have *K*_d_ in the micromolar range, but the complex with NADH is oxidized twice as fast. The most important thing is that quinones are reduced in a two-electron way, while nitroaromatics are reduced in a single-electron way with ~90% free radical yield. On the other hand, both groups of compounds share a common scattered parabolic (quadratic) dependence of log *k*_cat_/*K*_m_ on their *E*^1^_7_, which points to a possibility of a three-step (e^−^,H^+^,e^−^) reduction of quinones.

Mitochondrial apoptosis-inducing factor (AIF) is a FAD-containing 57–62 kD × 2 homodimer with NADH dehydrogenase activity, which is associated with the cytosolic side of the inner mitochondrial membrane [125]. The most established role of AIF is induction of caspase-independent programmed cell death, or apoptosis. Upon outer mitochondrial membrane permeabilization, the truncated fragment of AIF translocates to the cytosol and subsequently to the nucleus to trigger chromatin condensation and DNA degradation [125,126]. Importantly, the reduction of AIF by NADH leads to the formation of a dimer with a tight FADH^−^–NAD^+^ charge-transfer complex (*K*_d_ = 5–80 nM), which interacts less efficiently than the oxidized monomer. AIF is characterized by *E*^0^_7_ = −0.341 V, determined in the absence of NAD(H), which in the presence of NAD^+^ may rise up to −0.158–−0.176 V [126,127]. Quinones oxidize the FADH^−^-NAD^+^ complex of mouse AIF(Δ1–77), being reduced with 34% of single-electron flux. These reactions are very slow, their *k*_cat_ being lower than the maximal rate of FAD reduction, 0.31 s^−1^, and their *k*_cat_/*K*_m_ ranging from 1.2 × 10^4^ to 10 M^−1^s^−1^. The reactivity of quinones increased with an increase in their *E*^1^_7_ and decreased with their Van der Waals volume [128]. Due to the low reduction rate, it is unlikely that AIF is responsible for the lethal oxidative stress induced by menadione, as it has been suggested [129]. However, it is possible that reoxidation of reduced AIF by quinones, which prevents its dimerization, is an additional factor in their cytotoxicity.

**Scheme 2:**
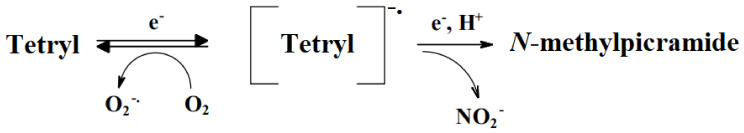
A scheme of the single-electron reduction of tetryl accompanied by its *N*-denitration [19].

Mammalian NAD(P)H:quinone oxidoreductase (NQO1, DT-diaphorase, EC 1.6.5.2) is a 2 × 30 kD homodimer containing one molecule of FAD per subunit. It was previously thought that NQO1 was localized in the cytosol and to some extent in the nucleus, but it has now been determined that 5–15% of it is found in mitochondria [130]. Among the physiological functions of NQO1, the maintenance of vitamins K1, K2, and K3 in their active reduced state, the protection of lipid peroxidation by the reduction of ubiquinone into its antioxidant form, and participation in the stabilization of transcription factor p53 are suggested [131]. The frequent increase in NQO1 activity in various tumors indicates a continuing interest in the development of NQO1-directed bioreductive drugs. Quinones and dicoumarol, competitive to NAD(P)H inhibitor, occupy the nicotinamide binding site [132]. The *E*^0^_7_ of rat NQO1 is equal to −0.159 V, and the maximal amount of red (anionic) FAD semiquinone is 8% at redox equilibrium. However, during the reoxidation of reduced NQO1, the transient semiquinone formation was not observed [133]. Rat liver NQO1 catalyzes two-electron reduction of quinones, with *k*_cat_ sometimes exceeding 1000 s^−1^; however, ArNO_2_ are reduced with much slower rates, with the final formation of hydroxylamines [134]. The exception here is tetryl (**18**), which is reduced by rat NQO1 in a predominantly single-electron way with *k*_cat_ = 73 s^−1^ and *k*_cat_/*K*_m_ = 2.6 × 10^5^ M^−1^s^−1^, with the formation of *N*-methylpicramide (Scheme 2) [134].

Rat NQO1 reduces tirapazamine (**45**) derivatives with 30% single-electron flux as well, but the reactions are slow, *k*_cat_ = 0.1–3.4 s^−1^ and *k*_cat_/*K*_m_ = 1.0 × 10^3^–1.3 × 10^4^ M^−1^s^−1^ [135]. The NQO1 inhibitor dicumarol decreases the cytotoxicity of these compounds.

### 3.5. Other Mitochondrial Flavoenzymes

The reduction of drugs or xenobiotics by most other mitochondrial flavoenzymes, leading to the formation of ROS, is partially investigated, without paying too much attention to the mechanisms of reactions and their characterization in quantitative terms.

Bovine heart succinate dehydrogenase (Complex II, SDH, EC 1.3.5.1) has covalently attached FAD with *E*_7_(FAD/FADH^•^) = −0.127 V and *E*_7_(FADH^•^/FADH_2_) = −0.031 V [136]. Reduced FAD transfers electrons to the chain of three FeS clusters with *E*^1^_7_ = 0.06–−0.26 V and *b*-type cytochrome with *E*^1^_7_ = −0.185 V, and further to ubiquinone [137]. SDH is not involved in the reductive activation of anthracyclines (*E*^1^_7_ = −0.33 V) [35], but it activates the soluble ubiquinone analogs (*E*^1^_7_ ~ −0.25 V), e.g., mitoquinone (**12a**) in bovine aortic endothelial mitochondria [38], and idebenone and decylubiquinone in mice hepatocyte mitochondria [138], which enhanced succinate-dependent O_2_ consumption. In line with this, yeast SDH is thought to be able to reductively activate 3-benzylmenadione derivatives of the antimalarial agent plasmodione [139]. It is possible that the sufficiently high redox potential of FAD may be a certain obstacle to mammalian SDH reactivity, but an additional factor is the shielding of the FAD isoalloxazine ring from the solvent by the *C*-terminal capping domain of the 68–70 kD subunit [140].

Another potentially important target for redox cycling drugs and xenobiotics in mitochondria is the fatty acid oxidation system, which consists of acyl-CoA dehydrogenases (CoADH), electron transfer flavoprotein (ETF), and ETF–ubiquinone oxidoreductase (ETF-QO). Pig liver mitochondrial general fatty acid CoADH has a FAD with *E*_7.1_(FAD/FAD^−•^) = −0.155 V and *E*_7.1_(FAD^−•^/FADH^−^) = −0.122 V, while the FAD of ETF is characterized by *E*_7.1_(FAD/FAD^−•^) = −0.014 V and *E*_7.1_(FAD^−•^/FADH^−^) = −0.036 V [141]. ETF-QO contains FAD and one Fe_4_S_4_ cluster, which are characterized by *E*_7.5_ (FAD/FAD semiquinone) = 0.028 V and *E*_7.5_ (FAD semiquinone/reduced FAD) = −0.006 V, while the Fe_4_S_4_ cluster is characterized by *E*_7.5_ = 0.047 V [142]. These sufficiently high redox potentials of flavoenzymes and FeS clusters may complicate the reduction of the compounds with low *E*^1^_7_ values. The data on the reduction of quinones by these flavoenzymes are scarce, and to the best of our knowledge, we have no information on the reduction of nitroaromatic compounds. CoADH was reported to be inactive towards several quinones with *E*^1^_7_ = 0.09–−0.24 V [143]. ETF does not reduce menadione and duroquinone; however, ETF-QO reduced them with *k*_cat_ = 18.1 s^−1^ and *k*_cat_/*K*_m_ = 9 × 10^5^ M^−1^s^−1^, and 6.6 s^−1^ and 4 × 10^5^ M^−1^s^−1^ [144]. This is several times slower than the reduction of various ubiquinones. However, it has not been determined to what extent these reactions are characterized by single-electron transfer.

## 4. Conclusions

This review attempted to systematize the known, but insufficiently abundant, data on the roles of mitochondrial flavoenzymes in the generation of free radicals of redox-active drugs and xenobiotics, and at least roughly estimate their ranking.

Among mammalian flavoenzymes, judging by the reactivity of the compounds, primarily by their *k*_cat_/*K*_m_, the most efficient free radical generator is the adrenodoxin reductase/adrenodoxin complex, where redox cyclers are reduced by ADX. In this case, the free radical yield reaches 100%, and the reaction follows the "outer sphere" electron transfer model. However, it should be emphasized that the role of ADR/ADX would only be important in specific cases where these proteins are abundantly expressed, e.g., in the adrenal cortex. The "classical" generator of free radicals of redox cyclers, isolated Complex I, has the highest reactivity among the described enzymes, except for ADR/ADX. However, in this case, the yield of Q^−•^ and ArNO_2_^−•^ is less than 100%. Furthermore, it is unclear to what extent external electron acceptors can divert electron flux away from the physiological oxidant ubiquinone, when Complex I is functioning normally in mitochondria. In this regard, the possibility that cytochrome *b*_5_ reductase/its complex with cytochrome *b*_5_ will be an equivalent free radical generator to Complex I cannot be ruled out. This possibility is based on the catalytic properties of this system, which, however, need more detailed examination.

Among mammalian flavoenzyme disulfide reductases, the isolated lipoamide dehydrogenase has a high quinone reductase activity, close to that of Complex I. Moreover, their amount in mitochondria is similar. However, this most likely does not reflect the LipDH functioning in the cell, where it shuttles between the oxidized and two-electron reduced forms. In this case, the activity of LipDH becomes close to the activity of other disulfide reductases, GR and TrxR, which in turn is about one order of magnitude lower than that of Complex I. Therefore, these enzymes should be treated as second rank free radical generators of redox cyclers. One reason for this is that in their EH_2_ form, the highest electron density is localized not on FAD, but on the thiolate. A separate case here would be the inactivation of TrxR by alkylating redox-active agents, after which this enzyme loses its antioxidant properties and becomes a prooxidant of limited activity.

The reactions of the flavoenzymes of the parasite mitochondrial respiratory chain with redox cyclers are largely uncharacterized, so it is unclear how important the single-electron reduction of ArNO_2_ by *P. falciparum* NDH2 is. Among other flavoenzymes, the high quinone reductase activity of *P. falciparum* TrxR is noteworthy, making this enzyme a potential target for redox-active antiplasmodial agents. LipDH isolated from *T. cruzi* mitochondrion also has high quinone reductase activity, similar to that of mammalian LipDH. However, for the same reasons as in the case of mammalian LipDH, it is not clear whether isolated *T. cruzi* LipDH functions in the same way as it does in the cell.

It is interesting to note that many of the flavoenzymes mentioned reduce quinones in a mixed single- and two-electron way, i.e., the yield of their free radicals is well below 100%. The reasons for this phenomenon have not been determined, but it can be stated that there is no relationship between the efficiency of single-electron reduction and the stability of the flavin radical and/or its degree of protonation. Another important point is the specificity of redox cyclers for particular flavoenzymes, as most data demonstrate only a roughly positive effect of *E*^1^_7_ on the reactivity of compounds. In order to increase the efficiency of mitochondrial redox cycling, it is necessary to study a wider range of compounds with different lipophilicity or electrostatic charge, as well as to focus on new targets, e.g., mammalian cytochrome *b*_5_ reductase/cytochrome *b*_5_ complex, the fatty acid oxidation system, as well as *P. falciparum* TrxR. Another approach would be to expand the research on mitochondria-targeted redox-active compounds of various structures, focusing primarily on their prooxidant activity.

## Figures and Tables

**Table 1:** Midpoint potentials of the single-electron reduction at pH 7.0 (*E*^1^_7_) of therapeutically or toxicologically relevant quinones, nitroaromatic compounds, and aromatic *N*-oxides or model compounds, and the p*K*_a_ of their free radicals [14,15,16,17,18,19].

No.	Compound	*E*^1^_7_ (V)	p*K*_a_
*Quinones*			
1.	1,4-Benzoquinone	0.09	4.1
2.	2-Methyl-1,4-benzoquinone	0.01	4.45
3.	2,3-Dichloro-1,4-naphthoquinone (dichlone) ^a^	−0.035	-
4.	2,6-Dimethyl-1,4-benzoquinone ^b^	−0.08	4.75
5.	5-Hydroxy-1,4-naphthoquinone (juglone) ^b^	−0.09	3.65
6.	5,8-Dihydroxy-1,4-naphthoquinone (naphthazarine) ^b^	−0.11	2.80
7.	9,10-Phenanthrene quinone ^c^	−0.12	-
8.	1,4-Naphthoquinone	−0.15	4.10
9.	2-Methyl-5-hydroxy-1,4-naphthoquinone (plumbagin) ^b^	−0.16	-
10.	2-Methyl-1,4-naphthoquinone (menadione) ^b^	−0.20	4.50
11.	9,10-Anthraquinone-2,6-disulphonate	−0.25	3.00
12.	Tetramethyl-1,4-benzoquinone (duroquinone)	−0.26	5.10
13.	Mitomycin C ^d^	−0.31	-
14.	1,8-Dihydroxy-9,10-anthraquinone ^b^	−0.325	3.95
15.	Adriamycin ^d^	−0.34	2.80
16.	1-Hydroxy-9,10-anthraquinone ^b^	−0.385	4,60
17.	2-Hydroxy-1,4-naphthoquinone ^b^	−0.41	4.70
*Nitroaromatic compounds*			
18.	2,4,6-Trinitrophenyl-*N*-methylnitramine (tetryl) ^e^	−0.191 ^f^	-
19.	4,3,6,7-Tetranitrobenzimidazolone ^e^	−0.197 ^e^	
20.	2,4,6-Trinitrotoluene (TNT) ^e^	−0.253	-
21.	5-Nitro-2-furaldoxime (nifuroxime) ^f^	−0.255	2.7
22.	1,4-Dinitrobenzene	−0.257	1.6
23.	4-(5-Nitrofurfurylidenamino)-3-methylthiomorpholine- 1,1-dioxide (nifurtimox) ^f^	−0.260	
24.	1,2-Dinitrobenzene	−0.287	2.2
25.	2-Nitrobenzimidazole	−0.300	
26.	4-Nitrobenzaldehyde	−0.325	
27.	2-Nitrofuran	−0.330	
28.	3,5-Dinitrobenzoic acid	−0.344	
29.	1,3-Dinitrobenzene	−0.348	2.4
30.	4-Nitroacetophenone	−0.355	2.6
31.	*N*-benzyl-2-(2-nitro-1*H*-imidazol-1-yl)acetamide (benznidazole) ^f^	−0.380	
33.	5-(Aziridin-1-yl)-2,4-dinitrobenzamide (CB-1954) ^d^	−0.385	
34.	1-Methoxy-3-(2-nitroimidazol-1-yl)propan-2-ol (misonidazole) ^g^	−0.389	
35.	2-Nitrothiophene	−0.390	
36.	5,5-Dimethyl-3-(4-nitro-3-(trifluoromethyl)phenyl)-2,4-imidazolidinedione (nilutamide) ^d^	−0.399 ^f^	
37.	1-(Methyl-2-nitro-1*H*-imidazole-5-yl)-methyl-*N,N*’-bis(2-bromoethyl)phosphorodiamidate (TH-302, evophosphamide) ^d^	−0.407	
38.	4-Nitrobenzoic acid	−0.425	2.8
39.	5-(Bis(2,2’-chloroethyl)amino)-2,4-benzamide (SN-23682) ^d^	−0.425	
40.	1-Methyl-2-((4-(methylthio)phenoxy)methyl)-5-nitro-1*H*-imidazole (fexinidazole) ^f^	−0.458 ^f^	
41.	5-Amino-3-nitro-1,2,4-triazole (ANTA) ^e^	−0.466 ^f^	
42.	Nitrobenzene	−0.485	3.2
43.	2-Methyl-5-nitroimidazole-1-ethanol (metronidazole) ^f^	−0.486	6.1
*Aromatic N-oxides*			
44.	1,2,4-Benzotriazine-1,4-dioxide	−0.318	
45.	3-Amino-1,2,4-benzotriazine-1,4-dioxide (tirapazamine) ^d^	−0.456	6.19
46.	3-Amino-1,2,4-benzotriazine-1-oxide	−0.568	
47.	Quinoxaline-1,4-dioxide	−0.575	

^a^ Fungicide; ^b^ naturally occurring quinone or its main functional moiety; ^c^ exhaust gas component; ^d^ anticancer agent; ^e^ explosive; ^f^ bactericidal or antiparasitic drug; and ^g^ radiosensitizer.

**Table 2:** Concentrations of flavoenzymes and their physiological redox partners relevant to the formation of free radicals of drugs and xenobiotics in mitochondria.

Protein	Source and Content	References
Complex I	Mice brain, 19 pmol/mg mitochondrial protein, bovine heart, 51 pmol/mg mitochondrial protein, mice brain astrocytes, 0.93 pmol/mg total protein ^a^	[24,25]
	Rat liver, 30% flavin content in the mitochondrial inner membrane	[26]
Adrenodoxin Reductase	Bovine adrenal cortex, 109 pmol/mg total protein ^a^	[27]
Adrenodoxin	Bovine adrenal cortex, 306 pmol/mg total protein ^a^	
Cytochrome *b*_5_	Rat liver, 390–240 pmol/mg protein of outer mitochondrial membrane	[28]
Lipoamide Dehydrogenase	Mice brain, 65 pmol/mg mitochondrial protein, bovine heart, 123 pmol/mg mitochondrial protein	[24]
	Rat liver, 10–17% flavin content in the mitochondrial inner membrane	[26]
Thioredoxin Reductase	Bovine adrenal cortex, 22 pmol/mg mitochondrial protein	[29]
Electron Transfer Flavoprotein	Amount in mitochondria of various rat organs (pmol/mg mitochondrial protein): 83 (liver), 63 (muscle), 60 (kidney), and 9 (brain)	[30]

^a^ The mass of proteins in mitochondria typically accounts for 5–10% of the total protein mass in the cell [26,28].

## Data Availability

No new data were created.

## Abbreviations

The following abbreviations are used in this manuscript:

ADR	NADPH:adrenodoxin reductase
ADX	Adrenodoxin
AIF	Apoptosis-inducing factor
ArNO_2_	Nitroaromatic compound
ArN → O	Aromatic N-oxide
b5R	NADH:cytochrome *b*_5_ oxidoreductase
CoADH	Acyl-CoA dehydrogenases
ETF	Electron transfer flavoprotein
ETF-QO	ETF–ubiquinone oxidoreductase
*E*^1^_7_	Single-electron reduction midpoint potential at pH 7.0
FAD	Flavin adenine dinucleotide
FMN	Flavin mononucleotide
GR	Glutathione reductase
GSSG	Oxidized glutathione
*k*_cat_	Catalytic constant
*k*_cat_/*K*_m_	Bimolecular reaction rate constant
LipDH	Lipoamide dehydrogenase
LipS_2_NH_2_	Lipoamide
NDH-2	Type-2 NADH:ubiquinone oxidoreductase
NQO1	DT-diaphorase
ROS	Reactive oxygen species
SDH	Succinate dehydrogenase
Trx	Thioredoxin
TrxR	Thioredoxin reductase

## Appendix A

**Table A1:** Midpoint potentials of single- and/or two-electron reduction of mitochondrial flavoenzymes and their metalloprotein redox partners relevant to the formation of free radicals of redox-active drugs and xenobiotics.

Protein	Redox Potential	References
*Dehydrogenases–electrontransferases and their redox partners*		
Complex I, bovine heart	*E*_7.5_ (FMN/FMNH^•^) = −0.415 V, *E*_7.5_(FMNH^•^/FMNH^−^) = −0.336 V	[32]
	FeS clusters: N1a, *E*^1^_7_ < −0.380 V; N1b, N3, N4, N5, *E*^1^_7_ = −0.20–−0.270 V; N2, *E*^1^_7_ = −0.050–−0.120 V	[33]
Succinate dehydrogenase, bovine heart	*E*_7_(FAD/FADH^•^) = −0.127 V, *E*_7_(FADH^•^/FADH_2_) = −0.031 V	[136]
	FeS clusters, *E*^1^_7_ = 0.06–−0.26 V; *b*-type cytochrome, *E*^1^_7_ = −0.185 V	[137]
Fatty acid CoA-dehydrogenase, pig liver	*E*_7.1_(FAD/FAD^−•^) = −0.155 V, *E*_7.1_(FAD^−•^/FADH^−^) = −0.122 V	[141]
Electron transfer flavoprotein	*E_7.1_*(FAD/FAD^−•^) = −0.014 V, *E_7.1_*(FAD^−•^/FADH^−^) = −0.036 V	[141]
Electron transfer flavoprotein–ubiquinone oxidoreductase	*E*_7.5_ (FAD/FAD semiquinone) = 0.028 V, E*_7.5_*(FAD semiquinone/reduced FAD) = −0.006 V, Fe_4_S_4_ cluster, *E*_7.5_ = 0.047 V	[142]
NADH:cytochrome *b*_5_ reductase, rat liver	*E*_7_(FAD/FAD^−•^) = −0.088 V, *E*_7_(FAD^−•^/FADH^−^) = −0.147 V (in the presence of NAD^+^)	[63]
Cytochrome *b*_5_, rat liver Mitochondria	*E*^1^_7_ = −0.107 V	[74]
NADPH:adrenodoxin reductase, bovine adrenal cortex	*E*^0^_7_ = −0.250–−0.274 V, *E*_7_(FAD/FADH^•^) = −0.320 V	[52,53,54,55]
Adrenodoxin, bovine adrenal cortex	*E*^1^_7_ = −0.260 V (free form), *E*^1^_7_ = −0.360 V (reductase-bound form)	
*Disulfide reductases*		
Glutathione reductase, human erythrocytes	*E*^0^_7_(E_ox_/EH_2_) = −0.227 V	[84]
Lipoamide dehydrogenase, pig heart	*E*^0^_7_(E_ox_/EH_2_) = −0.280 V, *E*^0^_7_ (EH_2_/EH_4_) = −0.345 V	[91]
Thioredoxin reductase, human mitochondrial	*E*^0^_7_ (EH_2_/EH_4_) = −0.270 V	[104]
*Other flavoenzymes*		
Apoptosis-inducing factor, mouse	*E*^0^_7_ = −0.341 V, *E*^0^_7_ =−0.158–−0.176 V (in the presence of NAD^+^)	[126,127]
NAD(P)H: quinone oxidoreductase (NQO1), rat liver	*E*^0^_7_ = −0.159 V	[133]
